# MicroArray Facility: a laboratory information management system with extended support for Nylon based technologies

**DOI:** 10.1186/1471-2164-7-240

**Published:** 2006-09-20

**Authors:** Paul Honoré, Samuel Granjeaud, Rebecca Tagett, Stéphane Deraco, Emmanuel Beaudoing, Jacques Rougemont, Stéphane Debono, Pascal Hingamp

**Affiliations:** 1 IPSOGEN SAS, Luminy Biotech Entreprises, 163 avenue de Luminy, Case 923, 13009 Marseille, France; 2 TAGC, INSERM ERM206, Parc Scientifique de Luminy, Case 928, 13288 Marseille Cedex 09, France; 3 Now at Swiss Institute of Bioinformatics, CH-1015 Lausanne, Switzerland; 4 Now at IGS, CNRS UPR 2589, 163 Avenue de Luminy Case 934, 13288 Marseille Cedex 09, France; 5 Now at CNRS – DSI, Tour Gaïa, rue Pierre-Gilles de Gennes, BP 21902, 31319 LABEGE CEDEX, France

## Abstract

**Background:**

High throughput gene expression profiling (GEP) is becoming a routine technique in life science laboratories. With experimental designs that repeatedly span thousands of genes and hundreds of samples, relying on a dedicated database infrastructure is no longer an option.

GEP technology is a fast moving target, with new approaches constantly broadening the field diversity. This technology heterogeneity, compounded by the informatics complexity of GEP databases, means that software developments have so far focused on mainstream techniques, leaving less typical yet established techniques such as Nylon microarrays at best partially supported.

**Results:**

MAF (MicroArray Facility) is the laboratory database system we have developed for managing the design, production and hybridization of spotted microarrays. Although it can support the widely used glass microarrays and oligo-chips, MAF was designed with the specific idiosyncrasies of Nylon based microarrays in mind. Notably single channel radioactive probes, microarray stripping and reuse, vector control hybridizations and spike-in controls are all natively supported by the software suite. MicroArray Facility is MIAME supportive and dynamically provides feedback on missing annotations to help users estimate effective MIAME compliance. Genomic data such as clone identifiers and gene symbols are also directly annotated by MAF software using standard public resources. The MAGE-ML data format is implemented for full data export. Journalized database operations (audit tracking), data anonymization, material traceability and user/project level confidentiality policies are also managed by MAF.

**Conclusion:**

MicroArray Facility is a complete data management system for microarray producers and end-users. Particular care has been devoted to adequately model Nylon based microarrays. The MAF system, developed and implemented in both private and academic environments, has proved a robust solution for shared facilities and industry service providers alike.

## Background

Transcriptome surveying using microarrays has become an established and widespread technique[1]. Although glass based microarrays and oligonucleotide chips are common in the gene expression profiling (GEP) landscape, Nylon supported microarrays coupled with radioactive detection, either home made [2-9] or from industrial suppliers [10-14], are an alternative still favored by some researchers[15], including the NIH's National Institute on Aging [16-19]. The resilience of this platform can be explained by the easy setup of this technical combination, its high sensitivity achieved without target amplification[20], and its cost effectiveness[16]. Technological development for this platform is ongoing, as demonstrated by a two 'isotope' dual channel variant that uses real time emission integration[21].

Regular increase in microarray reporter densities together with falls in unitary costs have meant experiments routinely generate tens of millions of data pieces to store, search and analyze[22]. For all but occasional microarray users, dedicated laboratory information management systems (LIMS) are now a requirement. Arguably one reason bench scientists are yielding to laboratory databases is increasing pressure from journal editors to have data appropriately submitted to international repositories as a prerequisite for publication[23].

Amongst the gene expression profiling LIMS that have been reported [24-34], glass dual channel Cy5/Cy3 and oligonucleotide chips are extremely well catered for. However, Nylon based microarrays are at best marginally supported by these software, with more substantial Nylon support only to be found in commercial products[35]. Drawing on experience with a previous LIMS[36], the specific functionalities that we have found critical for comprehensive Nylon based microarrays modeling include the ability to record so called 'vector' hybridizations (quantitations of spotted reporter amounts), the stripping and recycling of microarrays[37] and to model controls spiked into RNA samples [20,38]. Finally, the pharmaceutical industry manufacturing and regulatory rigor required for the development of diagnostic applications are typically not of major concern in freely available LIMS.

Here we report the development of MAF (MicroArray Facility), a LIMS designed to accommodate the following desiderata:

-stringent quality control, traceability and audit tracking to meet industrial requirements

-multi-platform support (i.e. Nylon, glass, and oligo-based microarrays)

-rich data annotation for MIAME standard compatibility[39]

-support for oncogenomic projects (i.e. clinical data)

-dynamic cDNA reporter annotation using public data banks (notably UNIGENE[40])

-MAGE-ML enabled[41]

-import robotic equipment files in native formats to reduce error-prone reformatting

-multi-user privilege environment to promote data sharing and ensure confidentiality.

The scope of MAF (as defined in [42]) is a local LIMS serving a community of researchers, such as found around academic shared facilities or commercial array and service providers. The MAF user interface is entirely web browser contained, thus there is no technical contra-indication to using MAF as a means to publish datasets over the Internet. However one-stop shops for gene expression data have innumerable advantages for public data mining[43]; thus we strongly recommend uploading public data to international archives such as ArrayExpress[44], CIBEX[45] or GEO[22] as a mechanism for publishing data.

MAF is a LIMS *sensu stricto *in that it records, tracks, structures, searches and reports all information required to establish gene expression profiles. High level downstream data analyses are carried out by exporting selected data to any of the myriad of dedicated analysis packages such as Cluster[46], BioConductor[47], MeV[26] or ProfileSoftware[48].

## Implementation

MAF follows a client-server architecture implemented as a web-based application, allowing simultaneous multi-user access to a central database. Client browsers connect to an Apache server in a Unix/Linux environment. The application is entirely written in Perl; since the Perl packages rely on the abstract DBI module, switching between different Relational Database Management systems (RDBMS) is as simple as changing a single line in the MAF configuration file. MAF has been implemented, tested and validated for the Oracle 8i and the PostgreSQL RDBMS. With Oracle 8i, certain Oracle-specific features such as backup tools and transportable table spaces can also be used. Having deliberately avoided RDBMS specific SQL syntax, we believe that MAF is easily portable to other SQL database platforms that have a Perl DBD driver.

The relational database underlying MAF – composed of 2258 fields held in 215 tables – is called ELOGE. Its schema extends the conceptual ArrayExpress design[44] which integrates microarray design and manufacturing, sample description, hybridization and data acquisition. Through a 5-year development cycle, ELOGE was considerably expanded to integrate fine grain modelling of wet lab routine procedures such as plate management, PCR quality control annotations, sequencing results, analytic validation, and GLP (Good Laboratory Practices) compliant protocols. The database scheme is designed to avoid computation-intensive queries and optimize user interface responsiveness.

**Figure 1:**
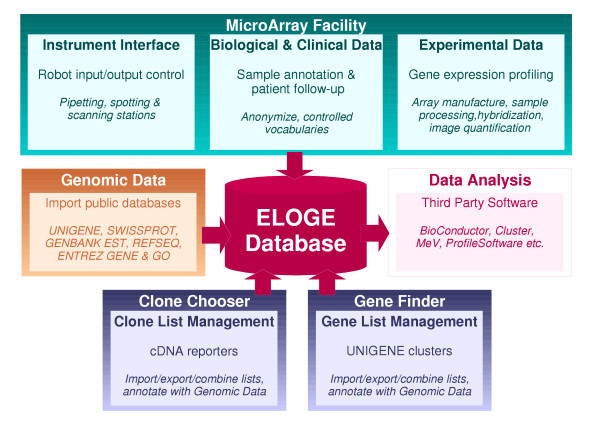
The Laboratory Information Management System (LIMS) is articulated around a relational database (ELOGE). Users manage gene lists, clone lists and microarray experiments through three software modules (Gene Finder, Clone Chooser and Microarray Facility respectively). Reporter annotation is achieved by importing genomic context data from public databases. Stored data can be queried and exported for analysis by appropriate third party software.

MAF is accompanied by two complementary software modules (Fig. 1): the Gene Finder (GF) and Clone Chooser (CC) which respectively manage gene and clone lists, as well as provide effective mapping from one to the other. Genomic data relevant to all three modules are imported into ELOGE every two months from several commonly used public databases (GENBANK EST, UNIGENE, SWISSPROT, ENTREZ GENE, GO and REFSEQ), allowing automatic and thorough annotation of genes and cDNA clones. Together, the MAF, GF and CC modules constitute a package called Discovery Software.

**Figure 2:**
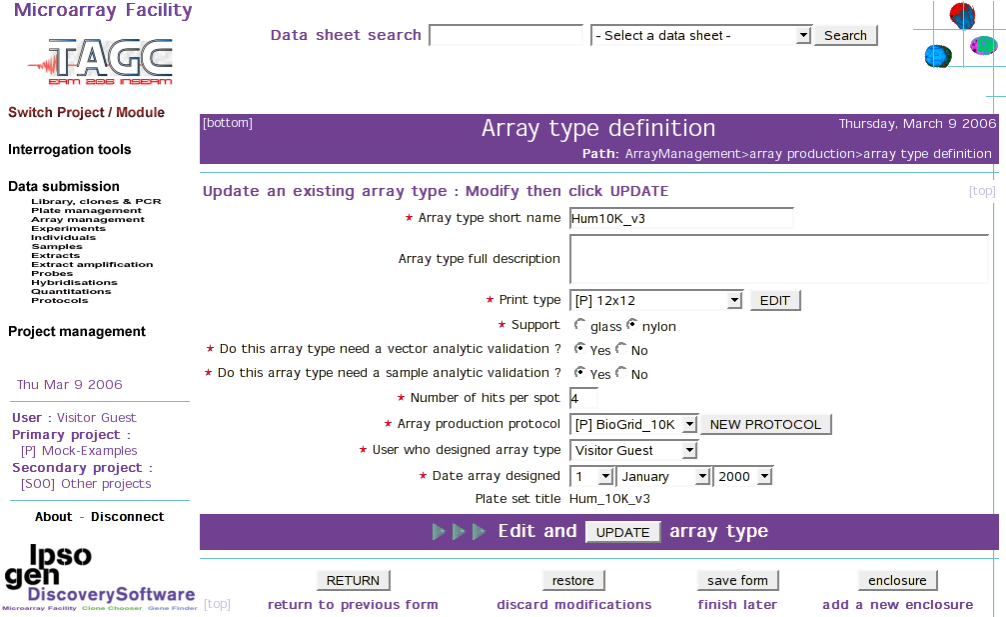
User data is collected through dedicated web form cascades. This example shows the Array Type definition form, which includes a "vector analytic validation" field specific to Nylon filters.

MAF user data are collected through web form cascades (Fig. 2). Where appropriate (e.g. array layouts or image quantitations) data files are uploaded using background queue processing to avoid tying-up the interface. Many instrument and third party software data files are thus directly imported using a large set of data formatting drivers (Table 1). MAF web forms can be used to add, update or view data. However, browsing MAF data is best achieved through a collection of hyper linked data reports specific for each step of GEP processing (Table 2).

Confidentiality is ensured through user login/password authentication and project containers. Access to a project's objects (arrays, plates, hybridizations, quantitations etc.) is controlled by the project owner through read, write, update, and delete independent privileges. Unique transaction ID's for each request, inactivity time-outs, enforced single user sessions and SSL network encryption complete the MAF security strategy.

## Results and discussion

### Clone library management

MAF clone management models the laboratory procedures for clone handling through wells, plates and plate sets. The LIMS precisely tracks clones from the resource plate to the array through replication, reorganisation, amplification and spotting steps.

**Figure 3:**
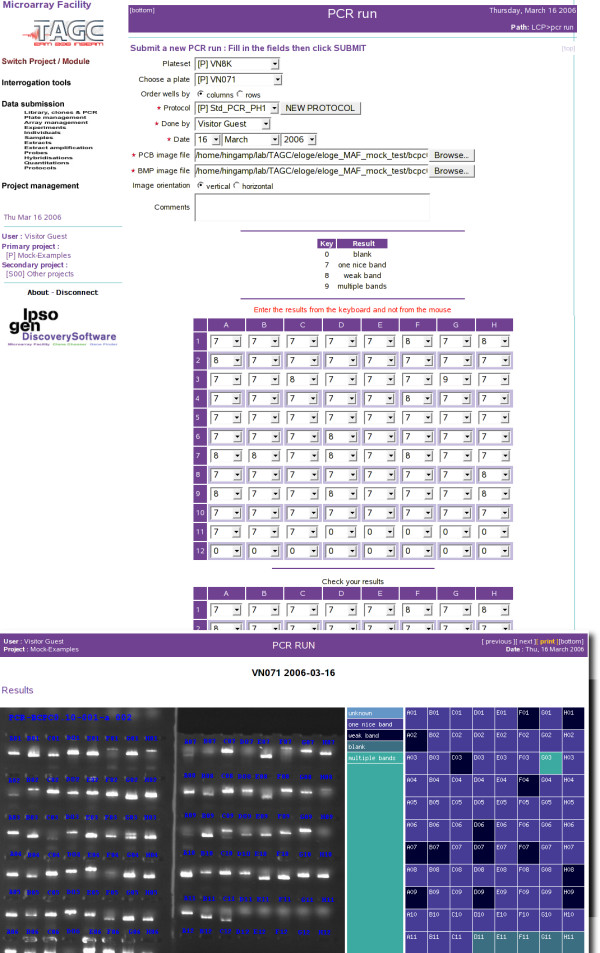
PCR run form used to verify cDNA clone amplifications on agarose gels. Double data submission (second table) is part of MicroArray Facility's quality control measures. Band readings are entered through keyboard strokes for better ergonomy and increased throughput.

Since plate handling is a cornerstone of GEP experimental work, much effort has been devoted to MAF's ergonomics and the minimization of manual data entry. For instance figure 3 shows the "PCR run" form used to enter quality control data from gel migrations of PCR amplifications. This synoptic side by side visualization of the gel picture with the colour coded annotated plate has allowed systematic verification of at least 10% of the plates before every plate set manipulation, without adversely affecting efficiency. Following this PCR quality control annotation, MAF can not only directly produce a reorganisation work list for the plate handling workstation (e.g. Tecan), but also verify and validate the reorganisation by comparing its original work-list with the trace file summarizing the work actually carried out by the work station.

Another example of MAF's routine quality controls is the update of sequence verified clone identities by monthly BLAST analysis and checking of clone to UNIGENE cluster associations.

### Microarray production

MAF manages every step of array production from abstract print type and array type definitions to batch production runs of physical arrays. A custom produced array design can be created by directly uploading layout definition files from spotting robots (e.g. Microgrid II or GMS), hence avoiding error-prone manual entry of the array design. Bypassing printing steps, microarrays or even oligo-chips from third party providers can also be loaded into MAF, albeit with less detailed annotation at the array design level.

In the case of Nylon based arrays, MAF manages the "vector probe hybridisation" post-production quality control. This step measures the quantity of reporter material bound at each spot through batch hybridisation of filters with a labelled oligonucleotide (the sequence of which is common to every spotted clone), followed by filter stripping.

Every array produced can be tracked down to the projects and hybridizations in which it has been used.

### Expression profiling data user submission

MAF data is partitioned into projects containing one or more experiments. An experiment ties together any number of hybridizations, usually all undertaken as part of the same experimental design. Experimental data submission follows the flow leading from biological samples to RNA extraction, labelling, hybridization, scanning, image feature extraction, and finally normalisation of measurements.

Feature extraction (image quantitation) results can be imported in a number of common software formats such as BZscan[49], ArrayGauge[50], Genepix[51], Imagene[52], and ProfileSoftware[48]. Where a format is not supported MAF provides a simple generic tab delimited format that can easily be produced with a spreadsheet program.

External controls added to labelled samples, such as RNA spike-ins which are commonly used with Nylon filters, are also quantitatively represented by MAF for a more accurate assessment of quality control and for spike based data set normalization.

MAF representation of biological samples links individuals, samples, RNA extracts and labelled extracts, each with a many-to-many cardinality. Rich quantitative and qualitative annotation of both individuals and samples is supported (e.g. code, age, mass, sex, tumor grade etc.), using either standard nomenclatures (such as oncology terms) or user defined parameters. This controlled vocabulary annotation of samples is of paramount importance for effective downstream correlation of expression profiles with experimental, biological and clinical factors.

### Data annotation and MIAME compliance

Results from high throughput gene expression profiling experiments differ from single gene measurements in that the effects of many more experimental parameters are likely to be observed. The proper correlation of expression signatures with biological parameters therefore requires careful recording of all known experimental variables.

This long recognized specificity of transcriptome analysis has led the Microarray Gene Expression Data Society (MGED[53]) to draw up MIAME, a set of minimal annotation guidelines for microarray based experiments[39]. All three international gene expression archives support MIAME standard data annotation, and an increasing number of scientific editors are requiring MIAME grade data for publication in their journals[23].

Thus MIAME compatibility has been a design ambition for the development of MicroArray Facility since its inception. This has directly impacted the underlying MAF data scheme as can still be seen in the [individual > sample > extract > labeled extract] part of the model. Defined name spaces for MIAME annotation are reserved in all relevant parts of the database relations. The annotation is either collected through web forms from the user (e.g. hybridization protocol), or generated automatically by MAF using imported public data (e.g. gene symbols from ENTREZ GENE or SWISSPROT).

Pivotal in MIAME is the requirement to attach laboratory protocols for all experimental steps. MAF has 15 protocol categories which are user supplied documents (e.g. text or PDF files) supplemented with optional or obligatory variable parameters, e.g. exposure time for "Image Acquisition" protocols.

Since software can only be 'MIAME supportive', i.e. potentially able to store the required annotation, MAF provides a MIAME check-list to help researchers make sure their data is actually MIAME compliant. The check list, accessible at any time, reports any missing annotation, in particular required protocols currently undefined in the project.

### Data export and interoperability

Data collected through the forms and their associated annotations can be viewed at any time through specific web reports launched from the permanent search box in the form header. All reports are dynamically hyper linked facilitating navigation across object categories. Displayed data can also be directly downloaded as tabulated text files. A more substantial reporting tool is also provided for more transversal data searches, such as finding all samples verifying specific criteria, for instance tumor grade or patient age. Search results are exported as classical flat file datasets including comprehensive sample and reporter annotations as well as the expression measurements. This text file format is compatible with most downstream analysis tools such as BioConductor, Cluster or ProfileSoftware [46-48].

MAF also currently provides an experimental MAGE-ML complete experiment export functionality comprising all MAGE packages (ArrayDesign, DesignElement, Experiment, BioAssay, BioMaterial, BioAssayData) suitable for exporting to data archives or to MAGE-ML enabled data analysis tools[47]. The MAF produced MAGE-ML has been validated and a test experiment was successfully pipelined into ArrayExpress.

### Regulatory compliance

Regulatory agencies are currently working to define a proper regulatory environment for GEP use in drug development and market approval processes. A guidance on Pharmacogenomic Data Submissions was issued by the FDA in March 2005[54]. This document defines the rules to be followed in order to ensure that GEP data submitted for drug approval will have the quality level required by the FDA.

Compliance with FDA 21 CFR part 11 regulations is audited at least once a year. The production version of MAF is currently 80% compliant with 21 CFR part 11. Ongoing developments aim to reach full MAF compliance with Good Laboratory Practices (GLPs). Guidelines for archiving records and standard operating procedures (SOPs) are distributed with the commercial version of the MAF (Discovery Software, see licensing).

## Conclusion

We have developed MicroArray Facility, a software tool for the management of microarrays which offers extended Nylon functionalities not found in other freely available LIMS (Table 3). All gene expression profiling steps from cDNA clone management to spot measurements are represented in the MAF database with annotation granularity compatible with MIAME.

Importantly, the MAF system has been tried and tested in both academic shared facilities and industrial environments managing cDNA and Affymetrix gene expression projects. Running in production for five years, MAF has established itself as a central information hub in the laboratory. Investment in data entry is rewarded by providing researchers with fast answers to common queries (e.g. "what is the expression profile of this new marker in our previously tested tumors?") and by helping extract more biological meaning from collected data.

## Availability and requirements

• **Project name: **MicroArray Facility (MAF)

• **Project home page: **

• **Operating system(s): **Platform independent

• **Programming language: **Perl, SQL

• **Other requirements: **Web Server (e.g. Apache), RDBMS (Oracle or PostgreSQL), MAGEstk

• **Licensing: **Royalty free, non-exclusive, non-transferable INSERM license for academics (excluding SOPs)

• **Any restrictions to use by non-academics: **Discovery Software license from IPSOGEN SAS

## Authors' contributions

PHi wrote the first draft of the manuscript. PHi, SDeb & SG conceived of the study, and participated in its design and coordination. PHo developed the MAF module. RT & SDer developed the CC and GF modules. EB and JR participated in MIAME/MAGE-ML implementation. All authors read and approved the final manuscript.

## Figures and Tables

**Table 1:** Equipment and third party software which are directly compatible with Microarray Facility.

**Operations**	**Software/Workstation**	**File format**
**Plate handling**	Tecan	Text

**Arraying**	Biogrid	MAF web form
	GeneMachines	Text
	Affymetrix 417 (ex GMS 417)	Text
	Microgrid II (Genomic Solutions)	CSV
	Generic	Tabulated text

**Image acquisition**	Raytest TINA (agarose gels) Generic	PCB + BMP BMP/TIF/IMG+INF

**Quantitation**	ArrayGauge 2.1 (Fuji)	Text
	BZScan 1.0 (INSERM)	Text
	GenePix 3.0 (Axon)	Text
	Imagene 4.0 (BioDiscovery)	Text
	ProfileSoftware Corporate/Cancer (Ipsogen)	Text
	Generic	Tabulated text

**Normalization**	ProfileSoftware Corporate (Ipsogen)	Text
	Generic	Tabulated text

**Enclosures**	Generic	Any (PDF, Text, DOC etc.)

Interfacing is through upload of output files in their native formats (except for the Biogrid arrayer which is modeled through a web form). Enclosures allow users to attach arbitrary files to any of the MicroArray Facility objects (such as spreadsheet results, publications, images etc.).

**Table 2:** List of objects that can be stored and queried from the MicroArray Facility web interface.

**Clone annotation**	**Vector hybridization**
Clones	Probe
Genes	Spike
Library	Oligo
Clone sequencing	Hybridisation
Clone history	Quantification result
PCR primer	**Extracts**
PCR run	Sample
Purified PCR run	Individual organism
**Plate management**	Parameter
Plate	Extract
Plateset	Extract amplification
Plate history	**Sample hybridization**
Plateset history	Experiment
Worklist	Probe
Freezer	Spike
**Array management**	Hybridisation
Array	Quantification result
Array batch	**Project**
Array analytic validation	Project
Array batch analytic validation	User
Strip	Process
Unstock	Enclosure
Production Follow-Up	**Protocol**
	Protocol

**Table 3:** Comparison of microarray LIMS with regard to licencing, specific technology support, regulatory compliance, MIAME compliance and Mage-ML export.

**Name**	**Supplier**	**Specific Support for**			**License Type**	**Regulatory Compliance**	**MIAME**	**Mage-ML export**
		**Nylon filters**	**2-color slides**	**Oligo chips**		**21 CFR part 11**		

ArrayDB 2.1	National Human Genome Research Institute (NHGRI)	*		*	OS	*	*	*
BASE 1.2	Lund University	±			OS	*		
GeneX Va/GEOSS 2.5.2	University of Virginia	*	±		OS	*		*
LIMaS 2	Medical Research Council (MRC) Harwell	*			RF	*		
MADAM	The Institute for Genomic Research (TIGR)	±			OS	*		
MADGE	University of Florida	*		*	RF	*	*	*
MARS 1.1.1	Graz University of Technology	*		*	RF	*		
maxdLoad2	University of Manchester	*			OS	*		
**MicroArray Facility**	Ipsogen/Institut National de la Santé et de la Recherche Médicale (INSERM)			±	RF			
MicroGen	Politecnico di Milano	*		*	OS	*		*
MiMiR	Imperial College London	*	*		RF	*		
QuickLIMS	German Cancer Research Institute	*		*	RF	*	*	*
SMD/Longhorn Array Database 1.5	Stanford University	*		*	OS	*		
Acuity 4.0	Molecular Devices	*			Com	*		
Expressionist	GeneData	*			Com			
GeneDirector 3	BioDiscovery	*			Com	*		*
GeneSpring GX Workgroup	Agilent technologies				Com			
GeneTraffic(Multi)	Stratagene				Com	*		
PARTISAN arrayLIMS	Clondiag				Com	*		
Resolver 6.0	Rosetta Biosoftware				Com			

OS: Open Source, RF: Royalty Free licence for academics, Com: Commercial Licence, 21 CFR part 11: aimed to comply with U. S. Food and Drug Administration Guidance for Industry [54], ± : partially supported feature, *: unsupported feature or feature not described in software associated documentation, website or publications.
